# HPO-driven virtual gene panel: a new efficient approach in molecular autopsy of sudden unexplained death

**DOI:** 10.1186/s12920-021-00946-7

**Published:** 2021-03-31

**Authors:** Ulrike Schön, Anna Holzer, Andreas Laner, Stephanie Kleinle, Florentine Scharf, Anna Benet-Pagès, Oliver Peschel, Elke Holinski-Feder, Isabel Diebold

**Affiliations:** 1 MGZ - Medical Genetics Center Munich, Munich, Germany; 2 Institute of Legal Medicine, Ludwig-Maximilians-University, Munich, Germany; 3 Department of Pediatrics, Technical University of Munich School of Medicine, Munich, Germany

**Keywords:** Sudden unexplained death, Molecular autopsy, HPO, Variant interpretation, Whole exome sequencing

## Abstract

**Background:**

Molecular autopsy represents an efficient tool to save the diagnosis in up to one-third of sudden unexplained death (SUD). A defined gene panel is usually used for the examination. Alternatively, it is possible to carry out a comprehensive genetic assessment (whole exome sequencing, WES), which also identifies rare, previously unknown variants. The disadvantage is that a dramatic number of variants must be assessed to identify the causal variant. To improve the evaluation of WES, the human phenotype ontology (HPO) annotation is used internationally for deep phenotyping in the field of rare disease. However, a HPO-based evaluation of WES in SUD has not been described before.

**Methods:**

We performed WES in tissue samples from 16 people after SUD. Instead of a fixed gene panel, we defined a set of HPO terms and thus created a flexible “virtual gene panel”, with the advantage, that recently identified genes are automatically associated by HPO terms in the HPO database.

**Results:**

We obtained a mean value of 68,947 variants per sample. Stringent filtering ended up in a mean value of 276 variants per sample. Using the HPO-driven virtual gene panel we developed an algorithm that prioritized 1.4% of the variants. Variant interpretation resulted in eleven potentially causative variants in 16 individuals.

**Conclusion:**

Our data introduce an effective diagnostic procedure in molecular autopsy of SUD with a non-specific clinical phenotype.

## Background

Sudden death (SD) of apparently healthy individuals is amongst the most challenging scenarios in clinical medicine. Sudden cardiac death (SCD) is the predominant cause of SD, with structural cardiovascular abnormalities often evident at autopsy. 10–30% of SD remain unexplained by conventional forensic autopsy procedures (the so-called sudden unexplained death, SUD). One-fourth of these autopsy-negative cases harbored an underlying variant that could explain the SD event [1–5]. Thus, molecular autopsy (post-mortem genetic testing) by high-throughput sequencing (HTS) technology represents an efficient tool to assess potential disease-causing mechanisms that remained undetected during conventional autopsy and may help to identify the cause of death and to detect families at risk for further SD [6–8]. Expert recommendation are available, emphasizing the importance of genetic testing [9–12]. However, identification of a causative variant in an individual who did not present with a specific clinical phenotype before the SUD is still challenging.

Since most of the reported likely causal variants were found in genes associated with cardiac ion channelopathies cardiomyopathies and metabolic disorders a fixed panel-based approach with a limited number and clinically well-defined genes is commonly used for identifying the genetic causes of SUD [1, 3, 4, 8, 13, 14]. Nevertheless, the number of SD predisposing genes is increasing consistently and the overall diagnostic yield of a fixed gene panel is limited. In comparison, whole exome sequencing (WES) has diagnostic power to identify potentially pathogenic variants also in rare causative genes, which have not been associated with SUD before and thereby elucidating novel pathomechanisms [15]. Unexplained SCD is often attributed to cardiac arrhythmia caused by cardiac ion-channel dysfunction, which is undetectable in a conventional autopsy. On the other hand, noncardiac conditions may also cause SD that is clinically indistinguishable from SCD. For example SUD in epilepsy is the most common cause of death related to epilepsy [16]. Thus, an obvious advantage of using an exome- based capture is that it opens the possibility of expanding the genes of interest without repeating the sequencing experiments.

For this reason, WES is increasingly used in clinical settings and represents the primary alternative to gene panel testing. However, data interpretation remains challenging because of a high incidence of variants of unknown significance (VUS) and the possible false assignment of variant pathogenicity. Rueda et al. compared a WES-based approach to a simulated gene panel for cardiac disease. The study found that exome-based analysis led to many likely pathogenic variants that could potentially explain SD [17]. However, due to the lack of robust databases for variants associated with SD, they could not make conclusive statements since the genes themselves have not been associated with SD.

As both WES and targeted panel sequencing yield accurate genetic diagnoses, clinicians are faced with the challenge of deciding which method to use. To improve variant interpretation in WES, the human phenotype ontology (HPO) was developed as a semantically computable international standardized vocabulary to capture phenotypic abnormalities in human [18]. Although, the number of HPO terms has grown substantially since the clinical integration and use of this ontology was established [19]. An obvious advantage of phenotype-driven filtering is that recently identified genes are automatically associated by the HPO term in the HPO database. Groza and coworkers developed a concept-recognition procedure that analyzes the frequencies of HPO disease annotations as identified in over five million PubMed abstracts by employing an interactive procedure to optimize precision and recall of the identified terms [20]. Here, we performed WES in tissue samples of 16 individuals with SUD after autopsy and provided a practical guide for filtering and prioritizing genetic variants by a HPO-driven virtual gene panel.

## Methods

### Samples and preparation

Autopsies on 16 SUD cases (9 adults, 23–53 years and 7 infants, 4 weeks to 9 months) were performed by forensic pathologists including general autopsy investigations, toxicology and histology. Cases were included if no specific cause of SD could be established at the medicolegal investigation. DNA samples for WES were extracted from post-mortem liver and/or heart tissue. Due to anonymization, co-segregation analyses of the variants were not performed. The study was approved by the ethics committee of the Medical Faculty at the Ludwig Maximilians-University Munich, Germany (Ethikkommission bei der Medizinischen Fakultät der Ludwig Maximilians-Universität München; Project Number: 20-1109).

### High throughput sequencing and bioinformatics pipeline

Whole exome sequencing (WES) of a custom capture kit (Agilent SureSelectXT) was carried out on an Illumina NextSeq 500 system (Illumina, San Diego, CA) using v2.0 SBS chemistry. Sequencing reads were aligned to the human reference genome (GRCh37/hg19) using BWA (v0.7. 13-r1126). SNV, CNV and INDEL calling on the genes was conducted using the varvis® software platform (Limbus Technologies GmbH, Rostock) subsequent coverage and quality dependent filter steps.

### Selection and use of human phenotype ontology (HPO) terms: creation of virtual gene panel

The HPO currently contains over 13,000 terms. Most ontologies are structured as directed acyclic graphs, which are similar to hierarchies but differ in that a more specialized term can be related to more than one less specialized term. The HPO terms used for variant filtering in our study were selected with the goal of covering phenotypic abnormalities that explain an unexpected sudden natural death. We selected the HPO term “arrhythmia” (HP: 0011675, associated with 356 genes), which belongs to the subclass *abnormality of cardiovascular system electrophysiology*. We selected the HPO term “sudden cardiac death” (HP: 0001645, associated with 72 genes) for variant filtering, which belongs to the category “cardiac arrest”. Since SUD is a fatal complication of seizures without recovery [21], we selected the specific HPO term “status epilepticus” (HP: 0002133, associated with 131 genes) which belongs to the category “seizure”. Since a lack of breathing may result in SD, we selected the HPO term “apnea” (HP: 0002104, associated with 266 genes) from the category “Abnormal pattern of respiratory”. Taken together, all cases of the study were annotated with the following set of HPO terms: arrhythmia, sudden cardiac death, status epilepticus and apnea. Overall, 672 different genes were associated with the selected HPO terms, thus creating a HPO-driven “virtual gene panel”.

HPO project data are available at http://www.human-phenotype-ontology.org. (Release: August 2020).

We additionally used the recently developed HPO similarity score (HPOSimScore; Release 12-2020) included in the varvis® software (Limbus Medical Technologies GmbH). The HPOSimScore calculates the phenotype similarity based on the selected HPO terms to improve efficiency of disease diagnoses (Phenotype/gene match). HPO score and match are controlled gene annotations which will be calculated after HPO terms have been associated to the case.

### Gene panel approach

To compare HPO-driven “virtual gene panel” with a fixed gene panel approach, additional data analysis was performed by a previously published multi-gene panel including 192 genes associated with SD based on the previous study from Neubauer et al. [8]. Genes of this panel were associated with cardiomyopathies, cardiac ion channelopathies, cardiovascular disease, connective tissue disorders, metabolic disease, respiratory disease and muscular dystrophy [8].

### Nomenclature, interpretation and classification of genetic variants

The nomenclature guidelines of the Human Genome Variation Society (HGVS) were used to annotate DNA sequence variants [22]. The functional consequence of missense variants was predicted with the amino acid (AA) substitution effect prediction methods SIFT (Sorting Invariant from Tolerated; http://sift.jcvi.org/), PolyPhen-2 (http://genetics.bwh.harvard. edu/pph2/), Mutation Taster, MAPP (http://mendel.stanford.edu/SidowLab/downloads/MAPP/index.html), GERP++ (http://mendel.stanford.edu/SidowLab/downloads/gerp/), Mutation Assessor (http://mutationassessor.org/r3/), FATHMM (http://fathmm.biocompute.org.uk/ fathmmMKL. htm), SiPhy (http://portals.broadinstitute.org/genome_bio/siphy/), PhyloP (https://ccg.epfl.ch/mga/hg19/phylop/phylop. html) and MetaLR (http://m.ensembl.org/info/genome/variation/prediction/protein_function.html#MetaLR). Splice-sites were predicted with MES (MaxEntScan; http://genes.mit.edu/burgelab/maxent/Xmaxentscan_scoreseq_acc.html) and SSF (SpliceSiteFinder; http://www.genet.sickkids.on.ca/~ali/splicesitefinder.html). Population databases were used to assess the allele frequencies of the variants: Database of all known Single Nucleotide Polymorphisms (dbSNP, https://www.ncbi.nlm.nih.gov/snp) and Genome Aggregation Database (gnomAD v2.2.1, https://gnomad.broadinstitute.org). The variants were classified according to the ACMG guidelines with the 5-tier classification system: class 5 (pathogenic), class 4 (likely pathogenic), class 3 (variants of unknown significance, VUS), class 2 (likely benign) and class 1 (benign) [23, 24]. The variant databases ClinVar (https://www.ncbi.nlm.nih.gov/clinvar/) and LOVD (Leiden Open-source Variant Database) were used.

## Results

### Specific selection of HPO terms added to variant assessment prioritizes 1.4% of the filtered variants

**Fig. 1:**
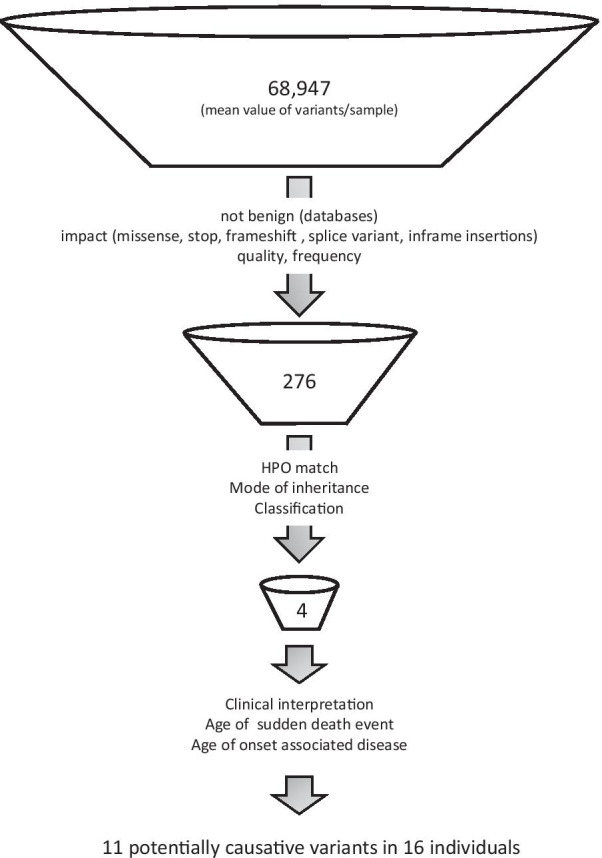
Flow chart of variant filtering for identification of potentially causative variants in SUD. Whole exome sequencing (WES) was performed in samples of 16 individuals who died suddenly and cause of death was not conclusive after a complete autopsy. Flow chart of variant filtering and the mean value of variants identified are shown. Overall, a mean value of 68,947 variants per sample were identified. The first step involved filtering by quality, population frequency, functional impact, LOVD and ClinVar classification to discard variants classified as likely benign/benign. This filter step resulted in a mean value of 276 variants per sample. Filtering by HPO matches (sudden cardiac death, arrhythmia, status epilepticus or apnea), mode of inheritance and variant classification, ended up in a mean value of four variants per sample. Clinical interpretation including the age of death resulted in eleven potentially causative variants

WES was performed in nine adults and seven infants with post autopsy unclear SD. In total, we obtained a mean value of 68,947 variants per sample (Fig. 1). The first filter step involved filtering by quality, allele population frequency, functional impact (missense, stop, frameshift, splice variants, in frame insertions) and variants not classified as likely benign and benign in different variant databases (ClinVar, LOVD). These steps resulted in a mean value of 276 variants per sample. The second filter step with the HPO-driven virtual gene panel (selected HPO terms: arrhythmia, sudden cardiac death, status epilepticus and apnea), variant classification and mode of inheritance prioritized a mean value of four variants per sample (1.4% of 276 variants). Interpretation of the identified variants in context with the age of the individual at the SUD event resulted in eleven potentially causative variants in 16 individuals (Table 1). Four variants were associated with the HPO term “arrhythmia”, seven with the HPO term “sudden cardiac death”, two with the HPO term “status epilepticus” and one with the HPO term “apnea” (Table 1). Interestingly, the majority of potentially causative variants was identified in infants. Six of seven infants carried at least one potentially causative variant, and three of nine adults carried at least one potentially causative variant.

**Table 1:** (a/b) Whole exome sequencing (WES) was performed in 16 individuals with SUD. The age of the individuals at the SUD event and the eleven potentially causative variants after stringent filtering process, name of the gene, OMIM, Reference Sequence (NM_number) of the gene, variant, amino acid (AA) change, variant type (splice_donor, splice_acceptor, missense), human phenotype ontology (HPO) match, population frequency (GnomAD), Single Nucleotide Polymorphism database number (dbSNP), MetaLR logistic regression (LR)-based score (MetaLrRank), ACMG criteria and classification are listed. Evidence of pathogenicity of each variant was shown. Pathogenic criteria: PVS1 (very strong), PM2 (moderate), PS3 (strong), PP3, PP4 (supporting). Evidence of benign impact: BS1 (strong). The variants were deposited and made publicly available at LOVD v.3.0 (Leiden Open Variation Database), LOVD variant link is listed

Age of SUD	Gene	*OMIM	NM_number	Variant	AA change	Variant type	HPO match	GnomAD	dbSNP	MetaLrRank	ACMG criteria	ACMG class	LOVD Variant link
*(a) List of identified variants after the described filter setting and HPO annotation*													
8 months	*DSG2*	125671	NM_001943.4	c.81+1G>C	p.(?)	Splice donor	Sudden cardiac death	–	–	–	PVS1-m, PM2	3	Individual #00329013 - Global Variome shared LOVD
3 months	*UPB1*	606673	NM_016327.2	c.917-1G>A	p.(?)	Splice acceptor	Status epilepticus	0.0017889	143493067	–	PVS1, PM2	4	Individual #00329092 - Global Variome shared LOVD
4 weeks	*SCN4A*	603967	NM_000334.4	c.787G>A	p.(Val263Ile)	Missense	Arrhythmia, Apnea	0.0000121	–	0.97394	PM1, PM2, PP3	3	Individual #00329093 - Global Variome shared LOVD
3 months	*RYR2*	180902	NM_001035.2	c.1939C>T	p.(Arg647Cys)	Missense	Sudden cardiac death	0.0001465	202040519	0.96504	PM2, PP3	3	Individual #00329094 - Global Variome shared LOVD
	*SCN8A*	600702	NM_014191.3	c.5392G>A	p.(Asp1798Asn)	Missense	Status epilepticus	–	–	0.92015	PM2, PP2	3	Individual #00329094 - Global Variome shared LOVD
9 months	*AKAP9*	604001	NM_005751.4	c.7096A>G	p.(Ile2366Val)	Missense	Sudden cardiac death	0.0000248	368823780	0.08032	PM2, BP4	3	Individual #00329095 - Global Variome shared LOVD
5 weeks	*SCN5A*	600163	NM_198056.2	c.3520C>T	p.(Arg1174Trp)	Missense	Arrhythmia, sudden cardiac death	0.0000348	367906630	0.9016	PM2, PP2, PP3	3	Individual #00329096 - Global Variome shared LOVD
28 years	*DTNA*	601239	NM_001390.4	c.1571G>A	p.(Arg524His)	Missense	Sudden cardiac death	0.0000906	142108185	0.50319	PP3, BS2	3	Individual #00329097 - Global Variome shared LOVD
32 years	*RAF1*	164760	NM_002880.3	c.1334T>G	p.(Leu445Arg)	Missense	Arrhythmia	0.0000239	–	0.95649	PM1, PP2, PP3, BS2	3	Individual #00329098 - Global Variome shared LOVD
23 years	*SCN5A*	600163	NM_198056.2	c.3152T>C	p.(Val1051Ala)	Missense	Arrhythmia, sudden cardiac death	0.000004	–	0.83793	PM2	3	Individual #00329099 - Global Variome shared LOVD
	*RBM20*	613171	NM_001134363.2	c.215A>T	p.(Asn72Ile)	Missense	Sudden cardiac death	–	–	0.80363	PM2	3	Individual #00329099 - Global Variome shared LOVD
*(b) List of additionally identified variants by the sudden death-gene panel*													
12 weeks	*CTNNA3*	607667	NM_013266.3	c.935C>T	p.(Ala312Val)	Missense	No	0.00002830	–	0.62663	PM2	3	Individual #00329100 - Global Variome shared LOVD
4 weeks	*KCNA5*	176267	NM_002234.3	c.98A>T	p.(Glu33Val)	Missense	No	0.0002093	71584818	0.93829	PM2, PP3	3	Individual #00329093 - Global Variome shared LOVD

To compare our findings by a HPO-driven virtual gene panel with those of a fixed gene panel approach, additional data analysis was performed by a sudden death (SD) gene panel according to Neubauer et al. [8]. The gene panel approach obtained a mean value of 4409 variants per sample compared to 68,947 variants per sample in WES. Same stringent filtering (except HPO-driven annotation), mode of inheritance and variant classification prioritized a mean value of three variants per sample. Interpretation of the identified variants in context with the age of the individual at the SUD event resulted in ten potentially causative variants in 16 individuals. Eight of the potentially causative variants were located in *AKAP9, DSG2, DTNA, RAF1, RANGRF, RYR2* and *SCN5A* (two SD cases) that have also been identified by the HPO-driven virtual gene panel (Table 1a). In addition, the gene panel approach identified potentially causative variants located in *CTNNA3* and *KCNA5* (Table 1b) that did not exactly match with the selected HPO terms but were annotated by the use of the HPOSimScore. The HPOSimScore of these genes were in the highest range of HPO-ranking, thus prioritizing those genes. The three potentially causative variants located in *UPB1, SCN4A* and *SCN8A* have not been identified by the SD gene panel (Table 1a). Eight genes (*CTF1, HCN2, GJD4, JLK, CALR3, MYOM1, PDLIM3, HEY2*) listed in the SD gene panel were not linked to any HPO term so far.

### Eleven potentially causative variants were identified in 16 individuals with post autopsy unclear sudden death by a HPO-driven virtual gene panel

Stringent filtering in combination with HPO annotation ended up in eleven candidate variants, three of them have not been identified before (Table 1). CNVs were not identified in any sample. Nine variants were missense and two splice variants. One of these splice variants were classified as likely pathogenic, one as VUS. The splice variant c.917-1G>A was found homozygous in a 3 months old infant in *UPB1*, annotated by the HPO term “status epilepticus”. The variant is listed in population-specific databases (rs143493067, gnomAD), and is classified as likely pathogenic/pathogenic in LOVD and with “conflicting interpretation” of pathogenicity in ClinVar. The other splice variant c.81+1G>C was found heterozygous in *DSG2* which was annotated by the HPO term “sudden cardiac death”. The variant was identified in an 8 months old infant and is not listed in population-specific databases, but at the same position a nucleotide change from G to T is listed in gnomAD (rs1237620145, gnomAD MAF: 0.003%). The rare truncating variant is located within the second exon of *DSG2* and is predicted to cause a splice donor malfunction. The variant was classified as VUS.

The nine missense variants were all classified as VUS (Table 1). Four variants in adults and four variants in children were located in genes that previously have been reported to be associated with cardiac channelopathies and cardiomyopathies, respectively. One variant was identified in *SCN4A* in a 4 weeks old infant and another in *SCN8A* in a 3 months old infant. Two individuals carried two VUS in different genes (Table 1).

## Discussion

A key challenge in using WES in molecular autopsy is finding the true causal variant among hundreds of rare variants. By filtering genes known to be associated with a particular HPO term, we shift the analysis focus from the entire exome to that part of the exome that is clinically interpretable in a diagnostic setting. We designed a HPO-driven virtual gene panel, and developed an algorithm that prioritized 1.4% of the variants by several filtering steps.

We compared our findings with a gene panel approach. Using the HPO-driven virtual gene panel identified three potentially causative variants in *UPB1, SCN4A* and *SCN8A,* that would have been missed by using the SD 192-gene panel [8] and another previously reported SD-gene panel [14]. On the other hand, gene panel approach identified two variants located in *CTNNA3* and *KCNA5* that do not exactly match the selected HPO terms. Importantly, the additional use of the HPOSimScore, which calculates the semantic similarity for HPO terms, shows high HPO-ranking for the variants in *CTNNA3* and *KCNA5*. These data indicate, that even if genes not exactly match to the selected HPO terms the additional use of the HPOSimScore is prioritizing potentially causative variants according to the HPO terms associated to patient and gene. Thus a phenotype-driven filtering by a selection of HPO terms in combination with the HPOSimScore is a very effective method to analyze WES data of SUD. The flexible HPO-driven assessment automatically prioritize recently identified genes, whereas the design of each targeted gene panel needs to be curated over time by adding new discovered genes to the already made panel. However, interpretation of variants identified in SUD cases is still challenging.

In total, we identified 13 potentially causative variants but only one variant was classified as pathogenic. The homozygous variant in *UPB1* was annotated by the HPO term “status epilepticus” and has been recently published to trigger seizures due to ß-ureidopropionase (UPB) deficiency in a recessive mode of inheritance [25]. Assmann et al. reported the same variant also homozygous in a 4 months old boy with an acute life threatening event (ALTE) with febrile status epilepticus [26]. The extent of the reduction in enzyme activity caused by a particular *UPB1* variant, along with other genetic and environmental factors may determine whether people with UPB deficiency develop neurological problems and the severity of these problems. Therefore, in many affected individuals with absent or mild neurological problems, the condition may never be diagnosed, and may thus explain that the identified variant has been found homozygous in one of 141,426 genomes from unrelated individuals. Importantly, epileptic seizures can induce malignant arrhythmias, possibly due to seizure-related effects on the autonomic nervous system [27]. However, the homozygous likely pathogenic variant in *UPB1,* recently associated to status epilepticus, has not been linked to SD before. Thus, a fixed gene panel-based approach consisting well-known genes linked to SD would have missed the variant in *UPB1*.

Beside the likely pathogenic variant in *UPB1*, we identified ten VUS. The majority of the VUS has been identified in genes previously having been reported to be associated with cardiac channelopathies (*SCN5A, AKAP9, RYR2*) and cardiomyopathies (*RBM20, RAF1, DSG2*) [7, 28–30]. One variant was detected in *DSG2* in an 8 months old girl. Pathogenic variants in *DSG2* are associated with arrhythmogenic right ventricle cardiomyopathy (ARVC), a disease that importantly predominantly affects adults in the 4th or 5th decade of life. If ARVC is diagnosed in the infantile stage, there should be clearly identifiable morphologic changes of the heart (fibrosis, dilation, fatty infiltration) before death occurs. Nevertheless, another study identified variants in *DSG2* associated with SUD in infants [31], indicating that interpretation of variants in context with the age of the individual at the SUD event is challenging.

One potentially causative variant was identified in *SCN4A* in a 4 weeks old infant. *SCN4A* variants are described as cause of autosomal-dominant myotonia and periodic paralysis [32]. Affected members developed in utero- or neonatal-onset muscle weakness of variable severity. In seven cases, severe muscle weakness resulted in death during the third trimester or shortly after birth [33]. Interestingly, variants in *SCN4A* have also been reported in patients with clinical diagnosis of Brugada syndrome, a primary arrhythmia syndrome [34]. Another potentially causative variant was identified in *SCN8A* in a 3 months old infant. Pathogenic variants in *SCN8A* have been associated with a wide spectrum of epilepsy phenotypes, ranging from benign familial infantile seizures to epileptic encephalopathies with variable severity [35].

Our data highlight that phenotype-based filtering could be used as complementary approach in particular to prioritize VUS by HPO-matches and HPOSimScore. Now, there are no forensic guidelines on the management and interpretation of VUS. Grassi et al. recently discussed the main elements and issues that differentiate the forensic management of cases in which VUS are found [36].

VUS will be found in many cases, and interpretation of the results would then require not only phenotypic information of the deceased but also family investigation to see whether the genotype segregates with a cardiac phenotype. If variants are proven as de novo, then this increases the potential utility. Our data further highlight that phenotype and genotype data should be used in conjunction to prioritize variants for further evaluation and may thus increase the overall solve rate especially in cases without specific clinical phenotypes like SD. In particular, HPO provides a structured, comprehensive and an international standard that could be used for developing algorithms and computational tools for clinical differential diagnostic in SUD [9].

Familial genetic testing should be performed to clarify potential pathogenic role of new variants and to identify genetic carriers that allows prevention of SD in relatives. Interpretation of rare variants, particularly those that are putatively pathogenic but lack functional and co-segregation analyses to support the predicted pathogenicity of the variant, is challenging. To date, many studies that used HTS identified putatively pathogenic variants in molecular autopsy but only a small number performed co-segregation analysis. Glengarry and co-workers reported that co-segregation studies are challenging to perform especially if the proband is an infant, due to difficulties in tracking families once a pathogenic variant which explains SD is found [37]. The lack of co-segregation analyses, together with absence of functional studies, results in many variants being classified as VUS, and their role within the SUD case remains speculative. Engagement of the family prior to genetic testing is essential to counsel for the possible uncertainty of the results and to permit family genotype–phenotype co-segregation studies.

Campuzano et al. demonstrated the value of co-segregation in SUD [38]. The presence of rare variants in asymptomatic family members aided the exclusion of some variants as being causative of the SUD. On the other hand, there might be significant variations in clinical features among family members due to variable penetrance und expressivity. In some cases, a phenotype may not be present. Reduced penetrance may result from a combination of lifestyle factors, environmental and genetics. This has significant implications because many apparently healthy individuals who harbor a pathogenic variant may not be prone to SD. We believe a comprehensive effort to collect and share genetic and phenotypic data is needed in order to define pathogenic variants more precisely and to provide quantifiable risks to living relatives.

## Conclusion

Molecular autopsy should be included in forensic protocols when no conclusive cause of death is identified. Prioritization of variants by a specific set of HPO terms could be used as a complement approach to perform a diagnosis in molecular autopsy. Identification of causative variants in molecular autopsy of SUD can allow prevention of SD in relatives.

## Data Availability

The variants identified and analysed during the current study are available in the LOVD database and the NCBI database and the accession numbers are listed in Tables 1a and 1b. By german federal data protection law (§ 3 Absatz 6 Bundesdatenschutzgesetz, BDSG, https://dejure.org/gesetze/BDSG/3.html) it´s not allowed to publish the raw sequencing data. The human reference genome (GRCh37/hg19) http://genome-euro.ucsc.edu/cgi-bin/hgGateway?redirect=manual&source=genome.ucsc.edu was used in our study.

## Abbreviations

ARVC: Arrhythmogenic right ventricle cardiomyopathy
GnomAD: Genome Aggregation Database
HPO: Human phenotype ontology
HPOSimScore: HPO similarity score
HTS: High-throughput sequencing
LOVD: Leiden Open-source Variant Database
MAF: Minor allel frequency
SD: Sudden death
SCD: Sudden cardiac death
SUD: Sudden unexplained death
SIDS: Sudden infant death syndrome
UPB: ß-Ureidopropionase
VUS: Variant of unknown significance
WES: Whole exome sequencing
